# A systematic simulation-based meta-analytical framework for prediction of physiological biomarkers in alopecia

**DOI:** 10.1186/s40709-019-0094-x

**Published:** 2019-04-04

**Authors:** Syed Aun Muhammad, Nighat Fatima, Rehan Zafar Paracha, Amjad Ali, Jake Y. Chen

**Affiliations:** 1 Institute of Molecular Biology and Biotechnology, Bahauddin Zakariya University, Multan, 60800 Pakistan; 2 Department of Pharmacy, COMSATS Institute of Information Technology, Abbottabad, 22060 Pakistan; 3 Research Center of Modeling and Simulation (RCMS), Department of Computational Sciences, National University of Sciences and Technology (NUST), Islamabad, 44000 Pakistan; 4 Atta-ur-Rahman School of Applied Biosciences (ASAB), National University of Sciences and Technology (NUST), Islamabad, 44000 Pakistan; 5 Informatics Institute, School of Medicine, The University of Alabama (UAB), Birmingham, USA

**Keywords:** Alopecia, Meta-analysis, Functional genomics, PPI networks, Biomarkers

## Abstract

**Background:**

Alopecia or hair loss is a complex polygenetic and psychologically devastating disease affecting millions of men and women globally. Since the gene annotation and environmental knowledge is limited for alopecia, a systematic analysis for the identification of candidate biomarkers is required that could provide potential therapeutic targets for hair loss therapy.

**Results:**

We designed an interactive framework to perform a meta-analytical study based on differential expression analysis, systems biology, and functional proteomic investigations. We analyzed eight publicly available microarray datasets and found 12 potential candidate biomarkers including three extracellular proteins from the list of differentially expressed genes with a *p*-value < 0.05. After expression profiling and functional analysis, we studied protein–protein interactions and observed functional associations of source proteins including WIF1, SPON1, LYZ, GPRC5B, PTPRE, ZFP36L2, HBB, PHF15, LMCD1, KRT35 and VAV3 with target proteins including APCDD1, WNT1, WNT3A, SHH, ESRI, TGFB1, and APP. Pathway analysis of these molecules revealed their role in major physiological reactions including protein metabolism, signal transduction, WNT, BMP, EDA, NOTCH and SHH pathways. These pathways regulate hair growth, hair follicle differentiation, pigmentation, and morphogenesis. We studied the regulatory role of β-catenin, Nf-kappa B, cytokines and retinoic acid in the development of hair growth. Therefore, the differential expression of these significant proteins would affect the normal level and could cause aberrations in hair growth.

**Conclusion:**

Our integrative approach helps to prioritize the biomarkers that ultimately lessen the economic burden of experimental studies. It will also be valuable to discover mutants in genomic data in order to increase the identification of new biomarkers for similar problems.

**Electronic supplementary material:**

The online version of this article (10.1186/s40709-019-0094-x) contains supplementary material, which is available to authorized users.

## Background

Alopecia or baldness is characterized by either patchy or complete loss of scalp hair [1]. It affects millions of men and women of all ethnic backgrounds and can be psychologically shocking. It is a complex polygenic disease that can occur at any age affecting the general population between 1 and 2% [1, 2]. The severity of the disease varies widely in the population, but very little is known about genetic and environmental factors. A comprehensive understanding of the physiological mechanisms and signaling pathways involved in the progression of the disease is lacking. How inflammation and infectious agents, genetic and immunological factors are associated with the pathophysiology of alopecia is not clear. The currently available treatment options have variable efficacy and are not satisfactory [3, 4] due to limited understanding of the causes and prognosis. The high prevalence and ineffective cure call for extensive research to establish better therapeutic strategies [5]. A systematic approach is required to identify candidate biomarkers regulating major biological pathways linked to alopecia and potential therapeutic targets for its therapy.

Biomarkers are quantifiable traits that can be used to analyze normal as well as pathological processes [6]. These candidate molecules can be used for prediction of relapse, screening and to examine the response of the treatment of hair loss. Due to a lack of a suitable analytical framework, significant problems have been confronted during the translation of candidate biomarkers from cDNA microarray research into proteomic investigations for clinical applications [7–9]. The gene expression profiling of the epidermal and dermal keratinocytes is responsible for the activation of adipogenic factors and the levels of chemical-markers [58] including WIF1, GPRC5B, PTPRE, and LMCD1. These biological molecules are known to regulate hair growth cycle. Recent progress in genomics and proteomics analysis enabled the identification of many proteins and the discovery of new biomarkers.

Microarray and quantitative proteomics generally produce a massive amount of data that need to be further examined to find biomarkers and drug targets. For genome-wide pathological analysis, numerous expression data sets are publicly available. Since data unavailability and platform diversity pose major challenges to the analysis of human diseases, the meta-analysis approach has received significant consideration for analyzing various datasets. This approach offers more detailed analysis, statistical support, and significant results compared to any single study [10, 11]. Simulation-based differential gene expression has enabled the discovery of genetic variants and the generation of a quantitative gene expression scoring system that is critical for disease pathogenesis. In this study, we designed a systematic and logical computational-based meta-analytical framework involving systems biology approaches that would be helpful in identifying the causative agents of hair loss leading to the discovery of biomarkers. Differential expression analysis of eight microarray datasets was performed to determine candidate genes with significant differential regulation scoring system. Identification of key proteins as potential biomarkers based on their expression patterns has become possible through the use of databases which reveal the expression of several genes in tissues [12]. Comprehensive in silico systems-level biological analysis was employed to determine significant alopecia biomarkers through a multistep prioritization method. The physicochemical and functional characteristics of these protein molecules were also studied. Moreover, the protein–protein interactions and pathways networks showed the functional association of our candidate proteins with hair follicle development and regulation of β-catenin, Nf-kappa B, cytokines, and retinoic acid levels. Probing of new biomarkers can deliver valuable insights in both biological and medical research after studying these molecular networks. It also increases biomarker discovery and can be used in large public datasets for better results in other diseases as well.

## Methods

### Identification and accession of eligible source data

The aim of the meta-analysis was to identify potential biomarkers for alopecia to effectively diagnose the causes of hair loss. Alopecia-related expression datasets were identified and retrieved from the Gene Expression Omnibus (GEO) database excluding the non-human studies. Information extracted from each dataset covers GEO accession number, sample type, platform, number of samples, and gene expression data. This study examines the genes commonly covered by hgu133plus2 chips. In these datasets, the Affymetrix Gene Chip Human Genome U133Plus2.0 Array platform and the annotation information (hgu133plus2) of probes were used to check differentially expressed genes. R-platform and BioConductor packages including Affy, AffyQCReport, AffyRNADegradation, AnnotationDbi, Annotate, Biobase, Limma, hgu133a2cdf and hgu133plus2cdf were employed to evaluate the statistical significance of the results (Additional file 1: Fig. S1).

### Normalization and differential expression analysis

Normalization was used to compare microarray data sets. The pheno-data files of these data sets were organized in an identifiable format [13]. Background correction, i.e. for perfect matches (*PM*) and mismatches (*MM*), was calculated as given in equation (i). Robust Multi-Array Analysis (RMA) was used to remove local artifacts and noise [14, 15].

$$PM_{ijk} = BG_{ijk} + S_{ijk} \left( {\text{i}} \right)$$

where, *PM* is a perfect match, Background (*BG*) caused by optical noise and non-specific binding (*S*); *ijk* is the signal for probe *j* of probe set *k* on array *i*.

$$BG\left( {PM_{ijk} } \right) \, = E\left[ {S_{ijk} |PM_{ijk} } \right] > 0$$

$$S_{ijk} \sim Exp(\lambda_{ijk} )BG_{ijk} \sim N(\beta i,\sigma^{ 2} )$$

where, PM-data is a perfect match (hybridization) representing combination of background (BG) and signals (S) of expression (E) for a set of probes (ijk), normalized (N) at default parameters. The signals of expressions were used to compute probe affinities. BioConductor “Array Quality Metrics” package was used to analyze the dataset that is normalized to the median expression level of each gene [14, 16, 17]. The expression value of a transcript having a *p*-value < 0.15 was considered as marginal log transformation and quantile normalization of arrays was used to bring them to the same scale. Gene–gene covariance matrices of each data set, ignoring the missing values, were calculated across all arrays (54675affyids). The formula for transformation is:

$$X_{norm} = F 2^{ - 1} \left( {F 1\left( x \right)} \right)$$

where, *F*1 and *F*2 are distribution functions of the actual and reference chips, respectively.

We used the RMA-algorithm to calculate averages between probes in a probe set in order to get a summary of intensities. BioConductor “AffyRNADegradation” package was used for RNA-degradation analysis and to measure the quality of RNA in these samples [18, 19]. In this study, we identified DEGs in each dataset from genomic experiments by pairwise comparison [20] and the Benjamini–Hochberg method was used for multiple testing corrections [21]. DEG sand duplicates pots along with the measurement of quality weights were shortlisted. The moderated statistics were calculated, genes were ranked and *p*-values were calculated. FDR < 0.05 (false discovery rate), *p*-value ≤ 0.05, Average Expression Level (AEL) ≥ 40% and absolute log fold change (logFC) > 1 were set as significant cut-off values [22].

### Gene overlap significance analysis

Common genes among ranked DEGs (*p*-value ≤ 0.05) of each microarray dataset included in this study were identified using Compare Two Lists (Bioinformatics & Research Computing, Whitehead Institute http://barc.wi.mit.edu/tools/).

### Subcellular localization and identification of secreted proteins

We predicted the subcellular localization of biomarker candidate proteins. We predicted the subcellular localization of alopecia-related significant DEGs using Signal P4.1 [23] and CELLO v.2.5 [24] to find the cellular compartments and categorize the selected proteins for effective applications. In most of the cases, the secretory and extracellular proteins of several cells are important sources of biomarker discovery as they indicate different states of the cells at a real-time [25]. These extracellular proteins that are secreted into the blood or plasma could be a convenient and easy way for patient clinical screening. The abnormal blood and plasma levels of such secretory proteins in various conditions would relate to the pathological conditions. Amino acid sequences as FASTA input format were used. SignalP4.1server was used to predict classical secretory proteins with D-value > 0.45 and SecretomeP2.0 server for non-classical secretory proteins with a neural network (NN) score = 0.5 [26]. Trans-membrane proteins were predicted using TMHMMv.2. with default options [27]. For biomedical text mining, the predicted genes were mapped with alopecia using the Comparative Toxicogenomics Database (CTD) to filter the information and related literature for the association of our selected differentially expressed genes with alopecia [28].

### Expression profile and functional enrichment analysis

We performed expression profile analysis based on average values of expression in each sample of each dataset in order to determine variations in gene expression among different sample sets [29, 30]. The functional annotation, gene ontology (GO) and pathway enrichment analysis [31, 32] of alopecia-related genes helped us to reveal biological functions and they were performed using the web-based FunRich [33], DAVID and Enrichr [34] annotation tools.

### Physicochemical and functional proteomic analysis

We analyzed the physicochemical properties of selected biomarker proteins by ExPASy ProtParam, ProtScale and Peptide Mass tools [35]. For empirical investigation and validation of the studies of two-dimensional (2-D) gels and mass spectrometry, these tools compute various physical and chemical parameters of proteins. ProtParam was used to calculate various physicochemical parameters, ProtScale for hydrophobicity analysis and Peptide Mass was used for calculating the masses of possible cleavable peptides and any known post-translational modifications. For further structural and functional analysis, PDBePISA interactive tool [36] was employed for the exploration of macromolecular interfaces. We identified all known motifs in our protein samples by Motif Scan web server [37]. Large proteins that make up structural and functional units have evolved by duplication of internal sequence repeats. Therefore, we identified gapped approximate repeats and short composition biased in protein sequences using RADAR web server [38, 39].

### Interactomic analysis

The protein–protein interactions (PPIs) reveal the topology and functional interaction of proteins that are useful to assess biological and pathological conditions [40, 41]. We can infer the functional relationship of proteins through genomic associations. In the PPI network, each protein is considered to have a relationship with one or more gene-sets connected with biological or molecular functions [42]. Functions of this biological network may show great differences of activity in the disease state as compared to normal. The functional interactors of source genes were retrieved with a high confidence score (0.999) from the STRING version-10 [43] and HAPPI databases [44]. For visualization and analysis of molecular networks, Cytoscape (version 3.2.1) was used [45] to identify the role of each seeder and target genes in alopecia and hair loss. In the network, the source nodes were categorized according to the degree of association with alopecia and any disturbance in the functionality of genes contributes to a disease phenotype. In Cytoscape, Network Analyzer was used to calculate topological properties of the network.

### Pathway-molecular mechanisms analysis in alopecia

The pathway analysis could be an essential tool for biomarker discovery to better understand the underlying biochemical mechanisms. KEGG, Reactome and Wiki pathways were used to curate and map the candidate biomarkers. We constructed an integrated metabolic network of alopecia-related potential biomarkers using PathVisio version-3 tool [46]. In this integrated network, the potential mechanism of each genetic marker in each pathway was studied based on data mining.

### Statistical analysis

We performed multiple testing to evaluate the significance of candidate biomarkers. Average expressions of genes between two phenotypic classes were compared, for gene *g*. The two-sample t-statistic was calculated. The false discovery rate (FDR) [21] is commonly used in the multiple testing of high-dimensional genomic data as a criterion for controlling false positives [47]. *FDR *= *V/R* is defined as the expected proportion of false positives among the genes declared significant. Since no null hypothesis was rejected, *V/R* is considered to be 0 when *R *= 0. The determination of the number of biological replicates is one of the current methods for controlling true positives. The variance was used to analyze the importance of differences between these groups. Data were expressed as mean ± SD. We performed all statistical analysis using the Limma-package of the R-platform version 3.1.3. The decision rule was set to *p*-value < 0.05.

## Results

### cDNA datasets and normalization

**Fig. 1:**
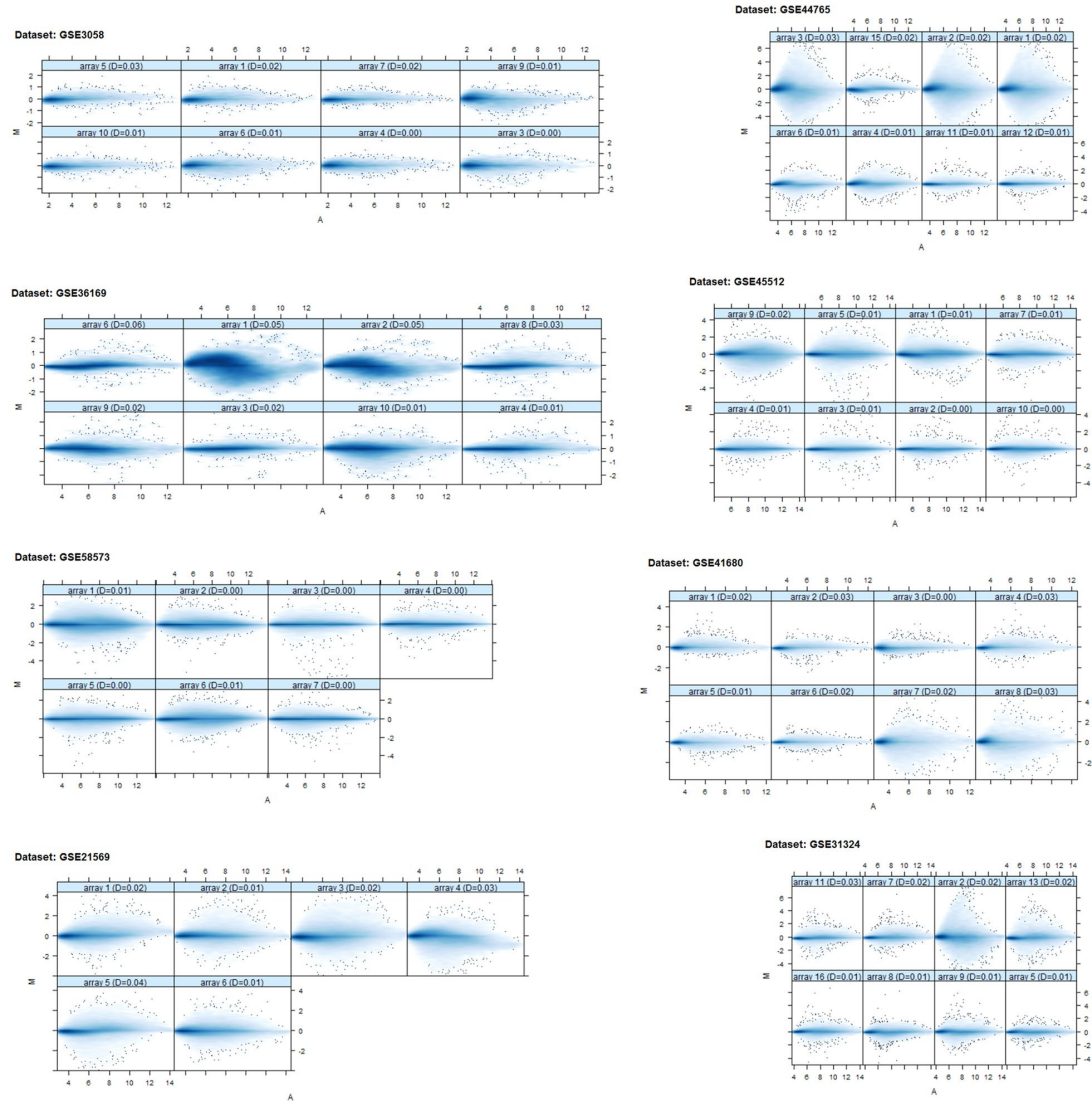
MA plots: individual array quality of each dataset after normalization. M = log_2_ (I_1_) − log_2_ (I_2_), A = 1/2 [(log_2_ (I_1_) + log_2_ (I_2_)], where I_1_ is the intensity of the array studied and I_2_ is the intensity of a “pseudo-array” that consists of the median across arrays. Typically, we expect the mass of the distribution in an MA plot to be concentrated along the M = 0 axis, and there should be no trend in M as a function of A

**Fig. 2:**
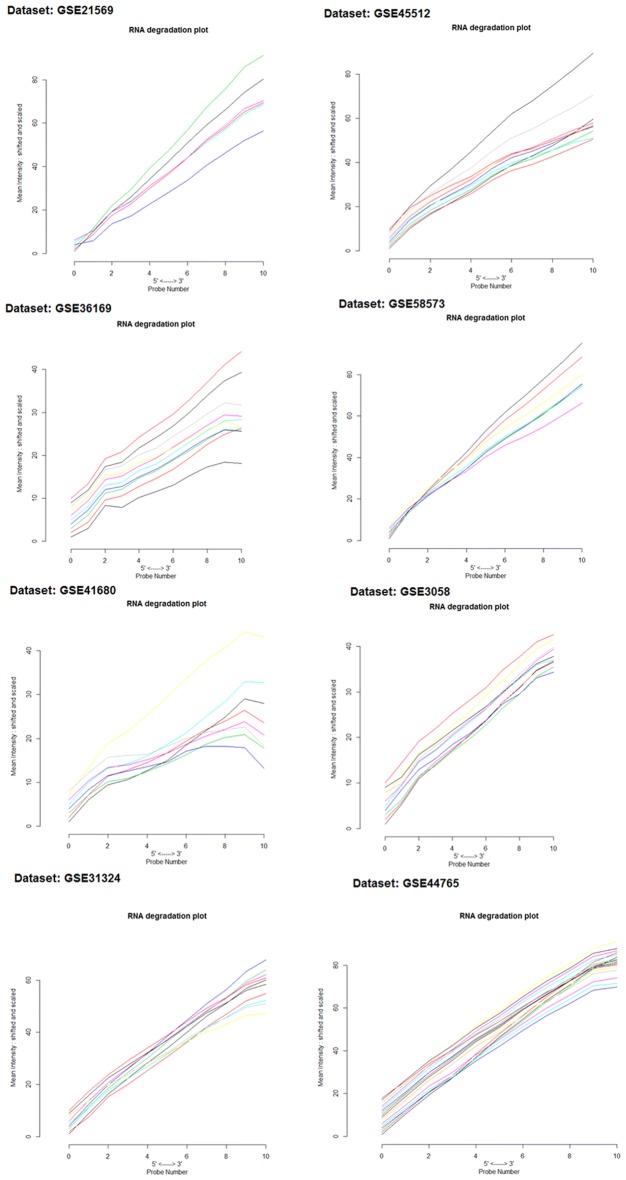
RNA degradation plot of each dataset: side-by-side plot produced by plotAffyRNAdeg representing 5′ to 3′ trend presenting an assessment of the severity of degradation and significance level

We accessed eight GEO datasets related to alopecia, hair loss, and scalp hair follicles cases. The AffyBatch object has the size of the arrays: 1164 × 1164, 732 × 732, 712 × 712, and 448 × 448 features with related Affyids (Table 1). Normalized distances between arrays of DNA chip showed the median expression level and the quantile normalization of the probes. The MA-plots indicated the quality of the individual array of each dataset after normalization (Fig. 1). The gene–gene covariance matrix across all arrays in each dataset by ignoring missing values was computed following a log-transformation of the arrays to make sure they were on the same scale. Decreased amount of RNA hybridization renders low-quality results and lowers the total signal strength. The intensity gradient 3′/5′ depends on the degree of competitive binding of specific and non-specific targets to a particular probe. The RNA degradation-plot of each dataset shows short probe sets near the 3′-end of the transcripts indicating that the 3′/5′ intensity gradient decreases by increasing degrees of saturation (Fig. 2). A single summary statistic for each array in the batch provides an assessment of the severity of RNA-degradation level (Additional file 2: Table S1). The list of tools, databases and software used in this study are shown in Additional file 3: Table S2.

**Table 1:** List of cDNA datasets

S. no.	Dataset accession no.	Total samples	Tissues	Species	Conditions/type	Platform	Size of arrays	AffyIDs	References
1	GSE3058	10	Head hair follicles	*Homo sapiens*	Men vs women	GPL201[HG-Focus] Affymetrix Human HG-Focus Target Array	448 × 448 features	8793	Kim et al. [66]
2	GSE21569	18	Scalp hair follicles	*Homo sapiens*	CD200-rich and CD34-positive	GPL570 [HG-U133_Plus_2] Affymetrix Human Genome U133 Plus 2.0 Array	1164 × 1164 features	54,675	Garza et al. [67]
3	GSE31324	16	Hair follicle dermal papilla	*Homo sapiens*	Micro-dissected DPs vs cultured DPs vs aggregated DPs	GPL571 [HG-U133A_2] Affymetrix Human Genome U133A 2.0 Array	732 × 732 features	22,277	Ohyama et al. [72]
4	GSE36169	10	Scalp epidermis	*Homo sapiens*	Bald vs haired	GPL96 [HG-U133A] Affymetrix Human Genome U133A Array	712 × 712 features	22,283	Garza et al. [68]
5	GSE41680	8	Keratinocytes	*Homo sapiens*	Case vs control	GPL571 [HG-U133A_2] Affymetrix Human Genome U133A 2.0 Array	732 × 732 features	22,277	Gazel et al. [71]
6	GSE44765	18	Hair follicle dermal papilla	*Homo sapiens*	Intact tissue vs cultured tissue vs spheroid culture	GPL570 [HG-U133_Plus_2] Affymetrix Human Genome U133 Plus 2.0 Array	1164 × 1164 features	54,675	Higgins et al. [69]
7	GSE45512	10	Areata skin	*Homo sapiens*	Case vs control	GPL570 [HG-U133_Plus_2] Affymetrix Human Genome U133 Plus 2.0 Array	1164 × 1164 features	54,675	Xing et al. [70]
8	GSE58573	7	Areata skin	*Homo sapiens*	Case vs control	GPL570 [HG-U133_Plus_2] Affymetrix Human Genome U133 Plus 2.0 Array	1164 × 1164 features	54,675	Xing et al. [70]

### Identifying DEGs and screening for potential biomarkers

**Fig. 3:**
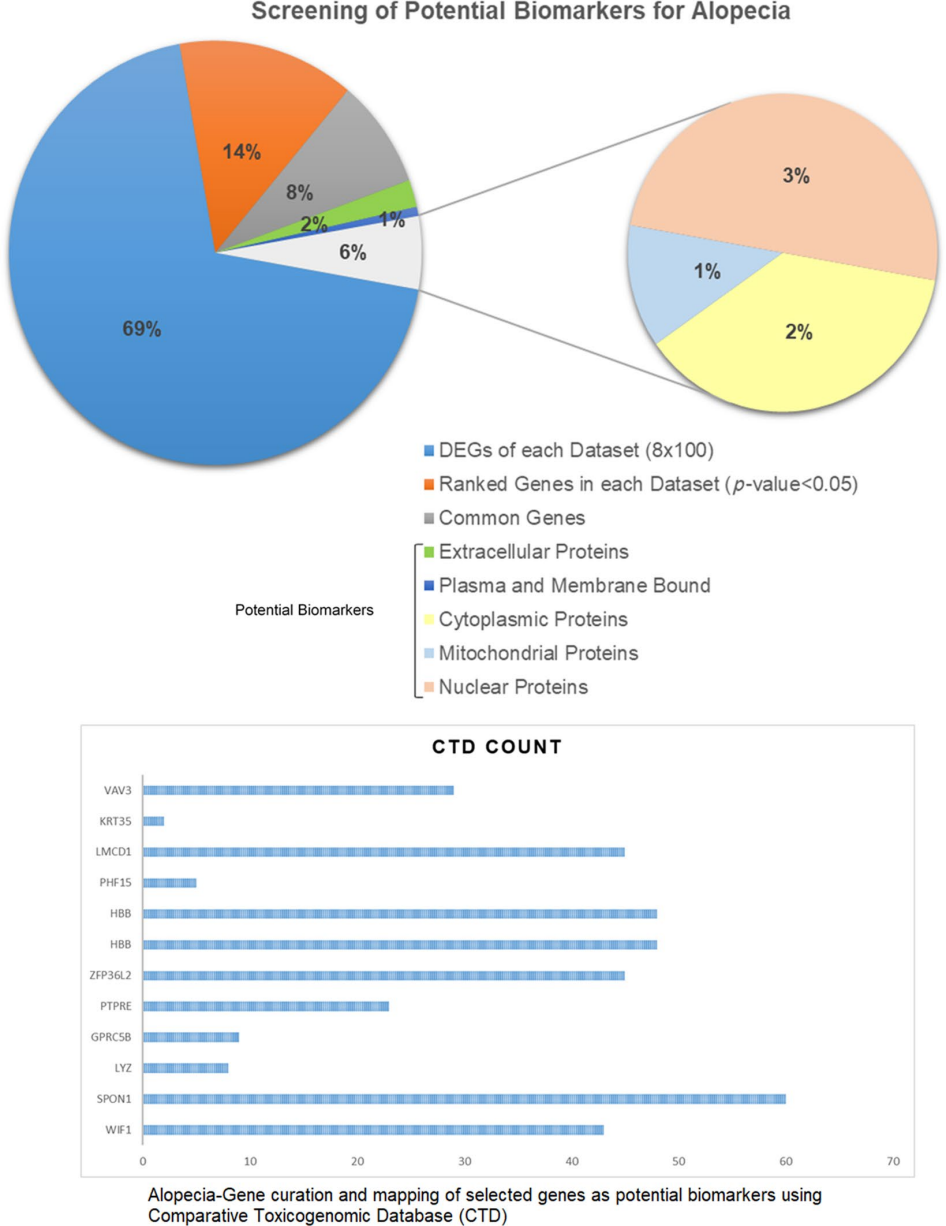
Screening steps to filter potential biomarkers for alopecia. The bar graph indicates the disease-gene mapping (alopecia-potential genes) using the comparative toxicogenomics database (CTD)

We identified a total of 100 DEGs in each microarray dataset by pairwise comparison between biologically comparable groups. Among these DEGs, the top 20 genes in each dataset were ranked and selected based on FDR (< 0.05), *p*-value (≤ 0.05) and logFC (> 1) parameters. From the list of ranked genes, 12 common genes (as potential biomarkers) of each dataset were identified (Additional file 4: Table S3). The sub-cellular localization of these genes indicates that WIF1, SPON1, and LYZ are extracellular, GPRC5B is membrane bound, PTPRE, VAV3, and HBB are cytoplasmic, ZFP36L2, PHF15, LMCD1, and KRT35 are nuclear while HBB is a mitochondrial-associated protein. We determined the transmembrane helices of these proteins based on a Hidden Markov Model (Additional file 5: Fig. S2). For disease-gene mapping, we found the role of these genes in alopecia and hair-loss and observed that SPON1 is the enriched term (Fig. 3).

### Expression profiling and functional enrichment analysis

**Fig. 4:**
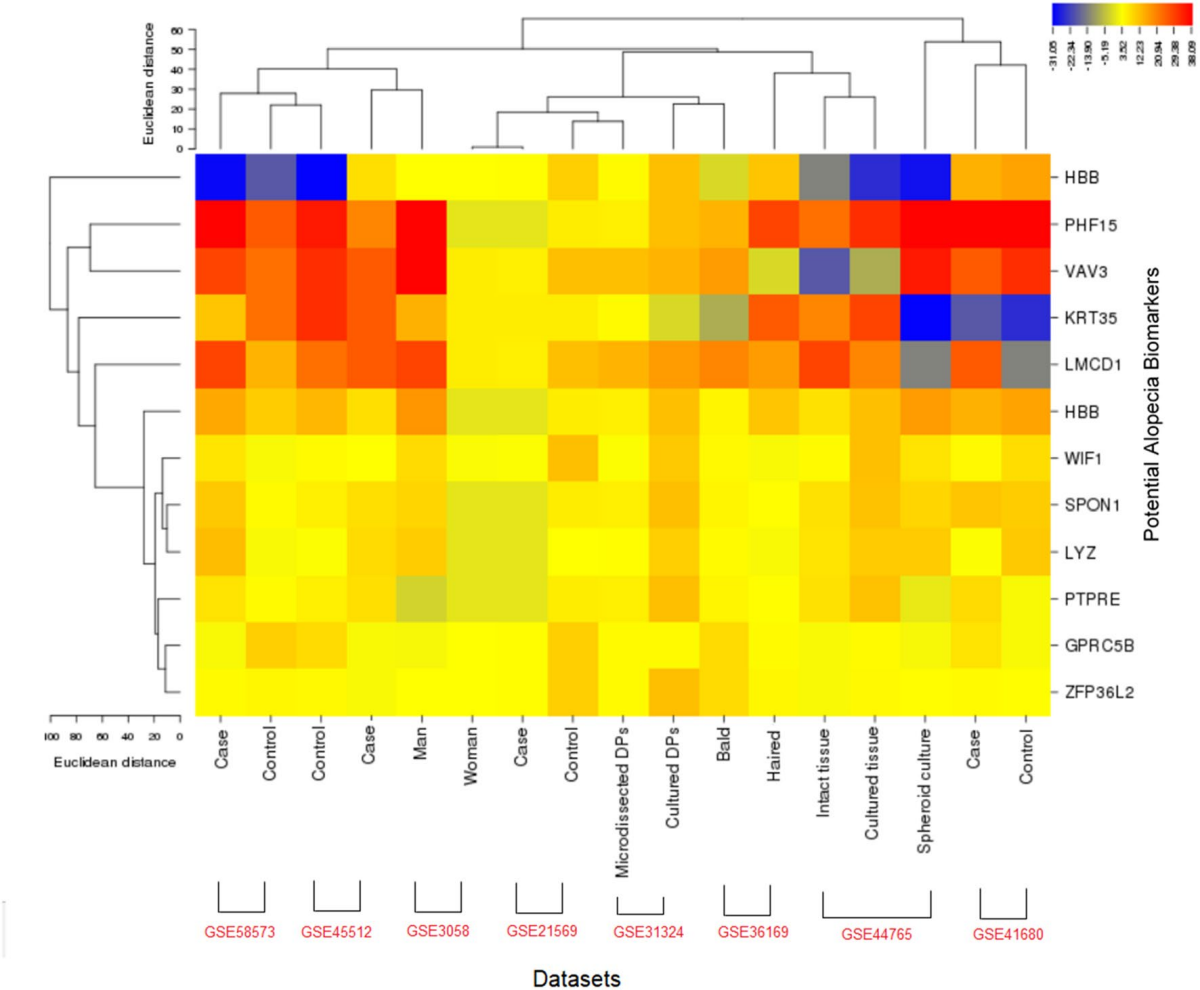
Expression profiling (indicates expression level) of potential biomarker candidates in each sample of each dataset

**Fig. 5:**
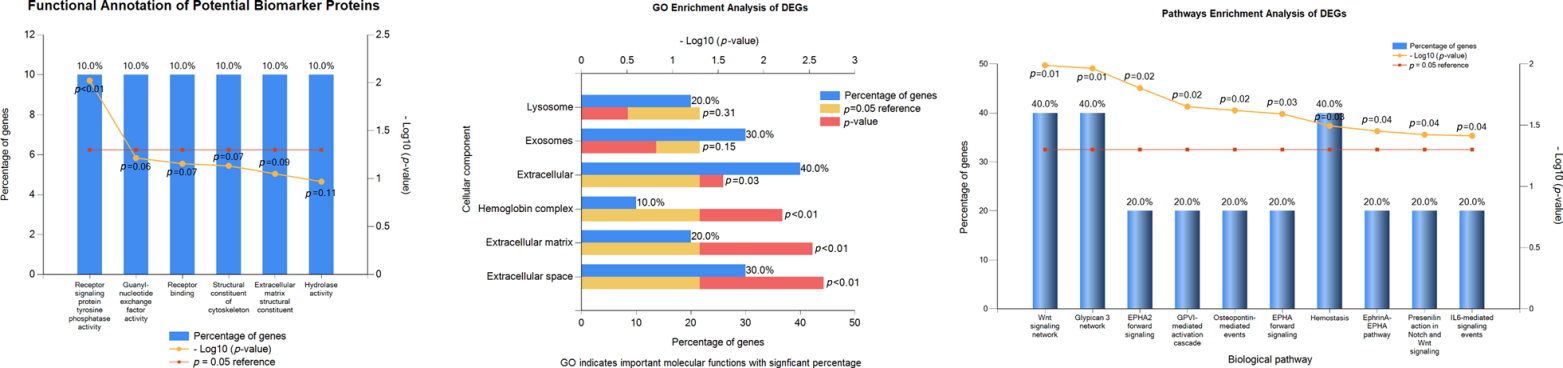
The functional annotation and GO analysis indicate important molecular functions of selected biomarker proteins using FunRich and DAVID annotation tools

We analyzed the expression level of 12 candidate biomarkers to observe the global picture of cellular functions between the biological samples of each dataset. This profiling showed the variation in expression levels and the relative activity of these genes in hair loss. In the heat map, the major variations in the expression levels of PHF15, KRT35, HBB, LMCD1, and WIF1 of each dataset are observed (Fig. 4). Pathway-enriched terms indicate a significant association of DEGs in biological pathways related to alopecia (Fig. 5). The pathway enrichment analysis of these genes uncovered important background information about cell growth, energy pathways, metabolism, and signal transduction (Table 2).

**Table 2:** Pathway enrichment analysis of potential biomarkers using Enrich tool

Pathway identifier	Pathway name	Entities found	Entities total	Entities ratio	Entities *p*-value	Entities FDR	Reactions found	Reactions total	Reactions ratio
R-HSA-3772470	Negative regulation of TCF-dependent signaling by WNT	1	15	0.00172	0.00686	0.05726	1	5	5.96E−04
R-HSA-5173214	O-glycosylation of TSR domain-containing proteins	1	39	0.00447	0.01776	0.05726	2	2	2.38E−04
R-HSA-977225	Amyloid formation	1	57	0.00653	0.02588	0.05726	2	14	0.001668653
R-HSA-5173105	O-linked glycosylation	1	104	0.01192	0.04684	0.05726	2	18	0.002145411
R-HSA-392499	Metabolism of proteins	2	916	0.10501	0.05726	0.05726	4	443	0.052800954
R-HSA-201681	TCF dependent signaling in response to WNT	1	198	0.02270	0.08775	0.08775	1	71	0.008462455
R-HSA-195721	Signaling by Wnt	1	294	0.03370	0.12815	0.12815	1	153	0.018235995
R-HSA-597592	Post-translational protein modification	1	416	0.04769	0.1775	0.17754	2	197	0.023480334
R-HSA-162582	Signal transduction	1	2411	0.27640	0.72584	0.72584	1	1693	0.201787843

### Identifying physicochemical and functional properties

Proteins possess different functional properties due to their dynamic physical, chemical, structural and amphiphilic nature. To explore the relationship of these properties, we investigated the physicochemical properties of selected potential biomarkers to facilitate the validation of studies using ProtParam, ProtScale, and Peptide Mass tools (Table 3). The total number of amino acid (aa) residues of biomarker proteins including WIF1 (379 aa), SPON1 (807 aa), PTPRE (700 aa) and LYZ (148 aa) showed a significant charge density while the hydrophobicity range at the C and N-terminus enabled an insight of the Îs-subunits of these proteins. The theoretical-pI indicated the solubility of these proteins at a given pH while the instability index showed that HBB and LYZ are more stable in vitro as compared to other proteins.

**Table 3:** Physicochemical properties of selected potential biomarkers using ProtParam, ProtScale, and PeptideMass tools

Potential biomarkers	Formula	Total amino Acids	Molecular weight	Theoretical pI	Ext. coefficient	Estimated half-life (h)	Instability index (II)	Hydropathicity (GRAVY)	Monoisotopic mass
WIF1	C_1813_H_2808_N_518_O_524_S_40_	379	415,27.80	7.84	46,920	30.0	54.70	− 0.264	41,499.78
SPON1	C_3929_H_6190_N_1114_O_1225_S_73_	807	90,973.40	5.85	149,205	30.0	61.34	− 0.604	90,913.59
LYZ	C_719_H_1146_N_220_O_207_S_11_	148	16,537.00	9.38	36,940	30.0	27.71	− 0.195	16,526.28
GPRC5B	C_2072_H_3244_N_526_O_542_S_23_	6407	44,933.13	8.06	65,930	30.0	42.45	0.52	44,903.60
PTPRE	C_3622_H_5651_N_979_O_1040_S_34_	700	80,641.69	6.57	97,010	30.0	35.24	− 0.342	80,589.99
ZFP36L2	C_2219_H_3438_N_650_O_702_S_19_	494	51,062.78	8.52	15,900	30.0	69.83	− 0.387	51,030.80
HBB	C_729_H_1128_N_196_O_202_S_4_	147	15,998.41	6.74	15,595	30.0	6.16	0.014	15,988.29
HBB	C_497_H_770_N_134_O_146_S_2_	101	11,022.51	6.03	12,490	7.2	1.52	− 0.239	11,015.64
PHF15	C_3798_H_6018_N_1078_O_1203_S_43_	789	87,408.65	5.15	92,360	30.0	57.12	− 0.639	87,353.08
LMCD1	C_1782_H_2822_N_502_O_539_S_29_	365	40,832.79	8.27	53,860	30.0	47.12	− 0.565	40,806.07
KRT35	C_2144_H_3464_N_620_O_715_S_31_	455	50,360.67	4.85	37,245	30.0	61.99	− 0.445	50,328.51
VAV3	C_3857_H_6055_N_1055_O_1148_S_35_	753	86,695.99	6.41	88,920	30.0	42.18	− 0.567	86,640.81

For functional proteomic analysis, we summarized and visualized the macromolecular interfaces of these proteins. The known amino acid residues of these proteins in the interface contact matrix are used to describe the molecular surface at the interface. The sequence motif indicated the pattern of biological significance involved in the shape of proteins and binding sites (Additional file 6: Fig. S3). We analyzed the motif sites of these proteins and identified the gapped approximate repeats and composition of residues for structural significance.

### Interactomic analysis

**Fig. 6:**
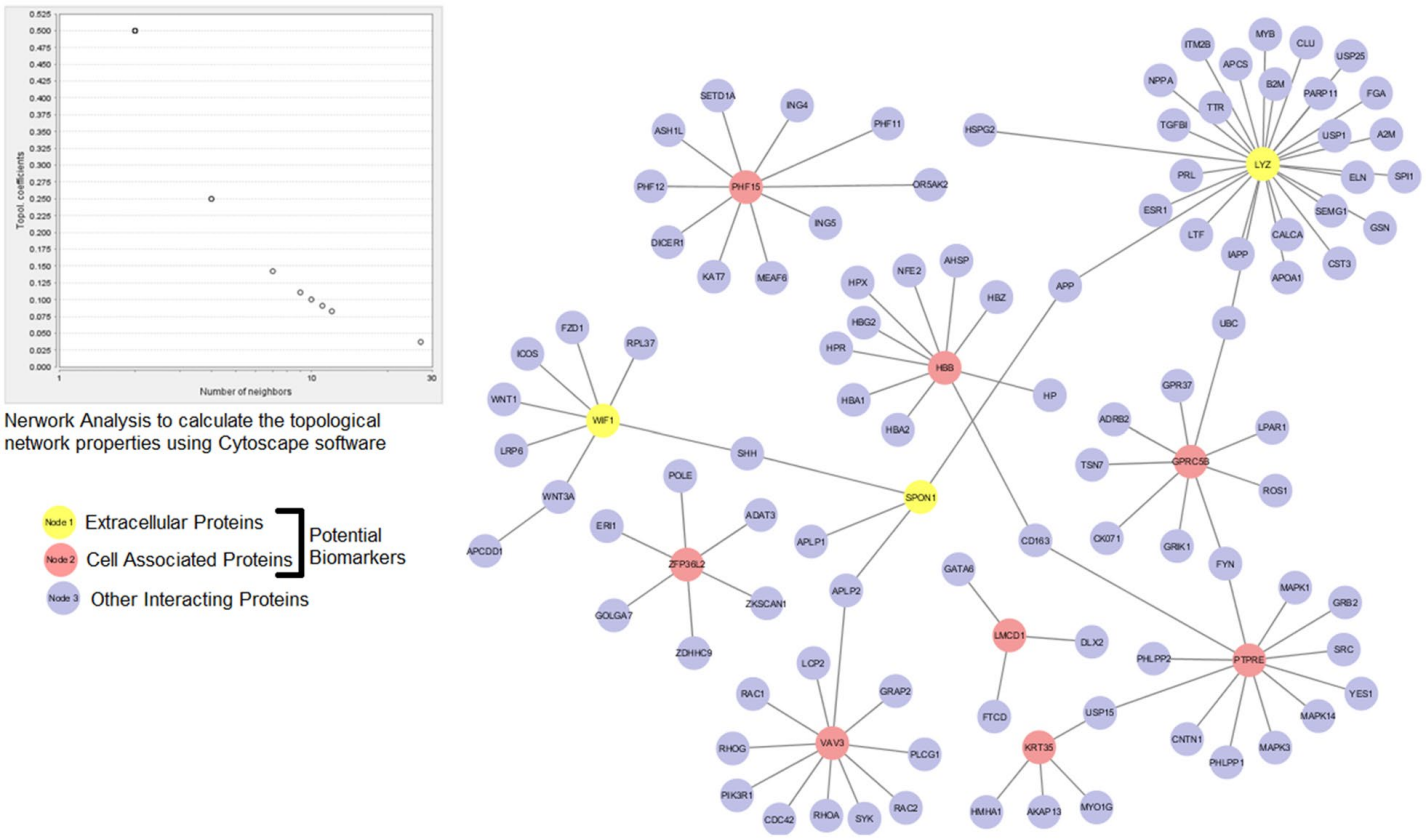
Protein–Protein Interaction Network. Interaction of seeder/source nodes (light yellow and red) with target nodes (light blue). The topological properties of the network were analyzed by Network Analyzer

To evaluate the topology and functional annotation of proteins, a protein–protein interaction (PPI) network was constructed. The network involved 111 nodes and 104 edges (nodes represent proteins and edges denote interaction), containing high scoring interaction partners (confidence score: > 0.9). The PPI network was largely categorized into three neighborhoods: light yellow and red nodes indicate the secretory and cell-associated potential biomarkers while the remaining blue nodes represent the other target proteins. We found that these candidate biomarkers interact functionally with other important protein targets including APCDD1 [48], WNT1, WNT31 [49], SHH [50], ESRI, TGFB1 [51] and APP [52]. This functional relationship shows the genomic connectivity of these proteins to disease phenotypes (Fig. 6). Of these proteins, SPON1 (spondin1, extracellular matrix protein) is principally interacting with SHH (sonic hedgehog protein facilitates androgen metabolic processes) and WIF1 (WNT inhibitory factor1) while WNT3A (Wnt-3a protein involved in WNT signaling pathway and hair development) is also interacting with WIF1. Similarly, PHF15 (PHD finger protein-15) is associated with the family of PHF11 and PHF12 contributing to clinical phenotype. We assessed the topological properties of the network using Network Analyzer to classify and optimize the network performance. After disease-gene mapping of these network genes using CTD, we observed more than 90 target genes having a functional connection with candidate biomarkers (source/seeder genes) in alopecia. Among them, RAC1, LPAR1, MAPK1, ITM2B, LTF are the enriched terms (Additional file 7: Fig. S4).

### Studying pathway models and associated mechanisms

**Fig. 7:**
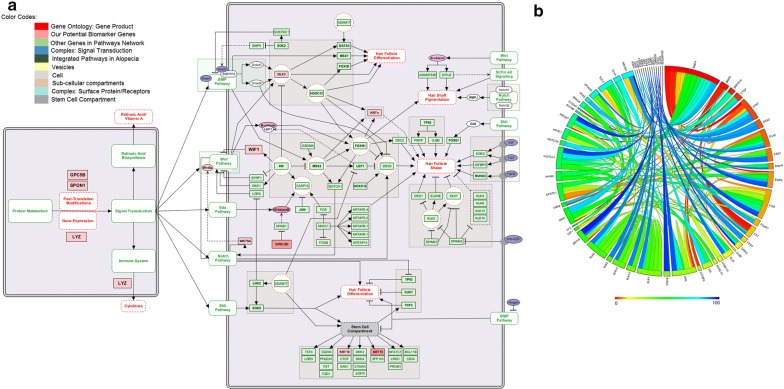
Reactomic analysis and molecular mechanisms in alopecia. **a** The role of integrated pathways in hair follicle differentiation, hair follicle shape, hair shaft pigmentation, and hair development. The pathways have been mapped using KEGG, Reactome and Wiki Pathways. Color codes are used to describe reaction steps of pathway model. **b** Circos Graph generated by Gene Terrain indicates the expression levels and the relationship of differentially expressed genes of each pathway

We studied the role of potential biomarkers in associated pathways to explore the molecular mechanisms of these molecules in alopecia and hair loss. These proteins are critically involved in the regulation of integrated pathways including protein metabolism, signal transduction, Wnt, BMP, Eda, Notch, and Shh pathways. The genetic ontology of these pathways is related to hair follicle differentiation, hair follicle morphogenesis and hair shaft pigmentation (Fig. 7a). Therefore, hair growth and shape depend on these pathways which interact with epithelial and mesenchymal cells. Integration of Wnt with the Eda pathway upregulates the expression of Shh ligand. The cascade of reaction is unified with BMP signaling and regulation of epithelial Notch expression [49]. The levels of β-catenin and Nf-kappa B [53], cytokines [35], retinoic acid [2] and the interaction cycle catalyzed by WIF1, SPON1, SHH, WNT3A, GPCR5, and LYZ are critical for modulating the signaling of Wnt, Shh, Notch and allied pathways. Regular expression patterns of these genes would affect the synthesis of these bio-molecules and dysregulation of these pathways lead to certain abnormalities of hair growth. In Fig. 7b, a circos graph represents the expression levels and interconnection of differentially expressed genes in alopecia.

### Statistical analysis

**Fig. 8:**
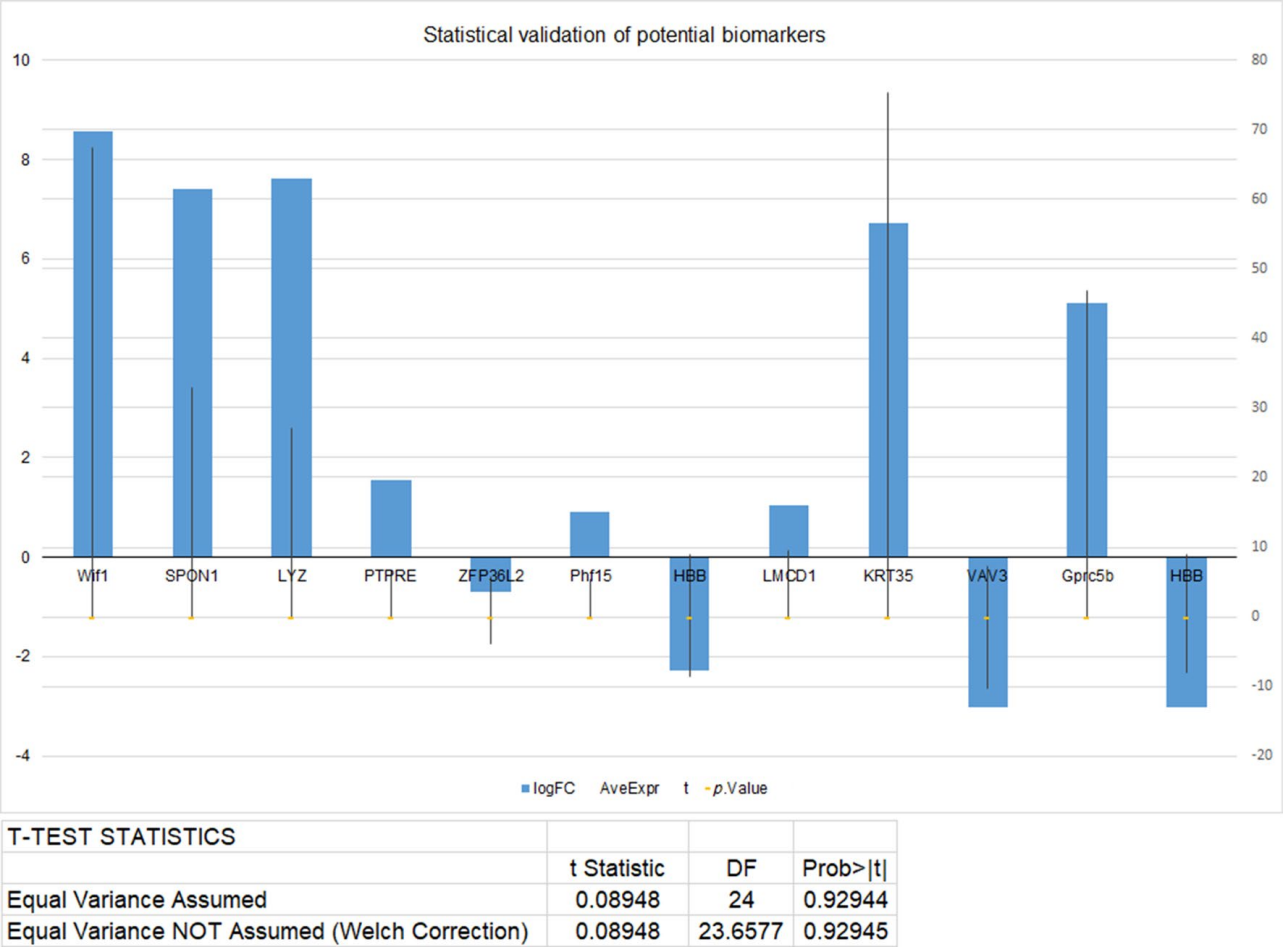
Statistical validation of potential biomarkers based on logFC, AveExpr, *p*-value and t-test statistics (classifier score)

We cross-validated our analysis by statistical algorithms. The significance of biomarker proteins in alopecia and hair loss between two phenotypes was tested based on logFC (> 1), Ave Expr (AEL ≥ 40%), *p*-value (< 0.05) and t-test statistics (Prob > |t|: 0.92944) (Fig. 8). We determined the false discovery rate (FDR) as a criterion for controlling false positives.

## Discussion

In recent years, the advances in technological developments have opened up a new era in systems biology, structural, functional and clinical proteomics. Specifically, functional proteomics helps us to understand the fundamental mechanisms of various pathophysiological states and reveals new opportunities for biomarker discovery. The current study critically explores the variations of gene expression levels in alopecia and hair loss. The meta-analysis provides a list of differentially expressed genes of scalp epidermis, hair follicle, dermal papilla and correlates with metabolic pathways. The expression profiling of these genes is indicative of the obvious differences among different tissue samples of clinical phenotypes.

We used various bioinformatics software, tools, and databases to systematically investigate expression data in order to discover specific protein biomarkers. The computational framework employed provides a logical step of the meta-analysis of cDNA microarray datasets associated with the exploration of DEGs. The steps implemented in this study are consistent and complement those technological pipelines described earlier [54, 55].

We filtered 12 common genes as potential biomarkers, namely WIF1, SPON1, LYZ, GPRC5B, PTPRE, ZFP36L2, HBB, PHF15, LMCD1, KRT35, HBB, and VAV3 (*p* < 0.05), from the list of 100 DEGs based on physicochemical and functional analysis. The dysregulation and functional role of these differentially expressed genes have also been studied in hair physiology [35, 49, 53]. In the protein–protein interaction network, we identified that these possible biomarkers are functionally associated with other interacting protein targets including APCDD1, WNT1, WNT31 [49], SHH [50], ESRI, TGFB1 [51] and APP [52]. The APCDD1 gene inhibits the WNT signaling pathway (WIF1), ultimately interfering with BMP, Eda, and Notch pathways, and this aberration causes alopecia and hair loss [48]. We observed more than 90 target proteins having a functional connection with candidate source genes in alopecia.

These candidate biomarkers are involved in the regulation of biological pathways including protein metabolism, signal transduction, Wnt, BMP, Eda, Notch, and Shh pathways [35, 53, 56]. The gene ontology (GO) is related to hair follicle differentiation, hair follicle morphogenesis and hair shaft pigmentation. Therefore, hair growth and shape depend on these important pathways that interact with epithelial and mesenchymal cells. WNT starts signaling through the signal mediator EDA/EDAR/NF-κB, i.e. nuclear factor kappa-light-chain-enhancer of activated B-cells, by upregulating Shh ligand expression. The pathway cycle is integrated with BMP signaling and upregulation of epithelial Notch expression [49]. Therefore, abnormalities of these pathways lead to irregularities of hair growth. The levels of β-catenin and Nf-kappa B are critical to modulate the signaling of WNT, SHH, and allied pathways [53]. Therefore, mutations in WIF1 would affect this level and hair growth. The cytokines [35] and retinoic acid are known to change skin and hair growth [2]. It has been observed that SPON1, GPC5B, and LYZ metabolize the proteins to synthesize retinoic acid and cytokines. The irregular expression pattern of these genes would change the normal level of these biomolecules affecting hair follicle function. The pathway modeling and integrated network-based analysis showed that these potential biomarkers are important for hair follicle development and variations in β-catenin, Nf-kappa B, cytokines and retinoic acid can be considered as diagnostic indicators of alopecia and hair loss. The protein–protein interaction network provides testable assumptions, albeit validation can only be justified through experimental studies.

The differential expression of WIF1 [57], PTPRE, HBB, and LMCD1 is associated with hair loss, morphogenesis, and alopecia. SPON1 and GPRC5B catalyze post-translational modifications and are integrated with WNT, Eda, Notch, Shh, and RAIG1 pathways. SPON1 is over-expressed in growing hair follicles [58, 59]. GPRC5 is associated with the differentiation of cells involved in the production of hard keratin and cortical cells of the hair shaft [59] and its dysregulation cause hair loss. LYZ-Ndufs4 showed a similar phenotype and disease conditions with significant attenuation of the alopecia [60]. Protein tyrosine phosphatase (PTPRE) and PHD finger protein 15 (PHF15) are differentially expressed in bald individuals responsible for androgenetic alopecia [61]. In animals, it has been reported that the down-regulation of ZFP36L2 gene that synthesizes a proline-rich zinc finger protein causes patchy alopecia [62]. The differential expression of LIM and cysteine-rich domains 1 (LMCD1) causes the aberration of Notch and Hedgehog signaling pathways leading to alopecia and hair-loss [63]. Under-expression of KRT35 and VAV3 genes level down the keratins and vav-3 guanine nucleotide exchange factor respectively, affecting the signaling pathways and hair growth [64, 65]. This study suggests that differential expression of these genes in both normal and mild state may be used as clinical biomarkers. We propose that the combination of several indicators may be more useful in diagnostic sensitivity and specificity instead of using a single biomarker.

## Conclusions

Systems biology studies of cDNA datasets helped us to identify potential DEGs of alopecia and hair loss. The methodological framework used revealed genome to phenome association in alopecia and hair loss. Our prioritization approach has found 12 potential physiological biomarkers of alopecia connected with other vital proteins including SHH and APCDD1. However, further molecular studies and research should be considered to authenticate the role of these genes in alopecia for effective treatment.

## Additional files

**Additional file 1: Fig. S1.** A framework of our study designed to identify physiological biomarkers in alopecia.

**Additional file 2: Table S1.** The function summaryAffyRNAdeg of Bioconductor package produced a single summary-statistic for each array in the batch dataset.

**Additional file 3: Table S2.** List of Databases, Software, and Tools used in this study.

**Additional file 4: Table S3.** Preliminary investigation of common and ranked differentially expressed genes of each microarray dataset.

**Additional file 5: Fig. S2.** Prediction of transmembrane helices in selected potential biomarker proteins using TMHMM Server v. 2.0.

**Additional file 6: Fig. S3.** Structural and functional properties of potential biomarker candidates. Studies of macromolecular interfaces using PDBePISA interactive tool. Motif scan in protein sequences were studied using Motif tool. Identified gapped approximate repeats and complex repeat architectures using RADAR (Rapid Automatic Detection and Alignment of Repeats) tool.

**Additional file 7: Fig. S4.** Data mapping: The role of differentially expressed genes in alopecia was mapped using the Comparative Toxicogenomics Database (CTD).
