# Supplementary figures and images for: A systematic simulation-based meta-analytical framework for prediction of physiological biomarkers in alopecia
Source: J Biol Res (Thessalon). 2019 Apr 4;26:2. doi: 10.1186/s40709-019-0094-x (PMC6449998; doi:10.1186/s40709-019-0094-x)

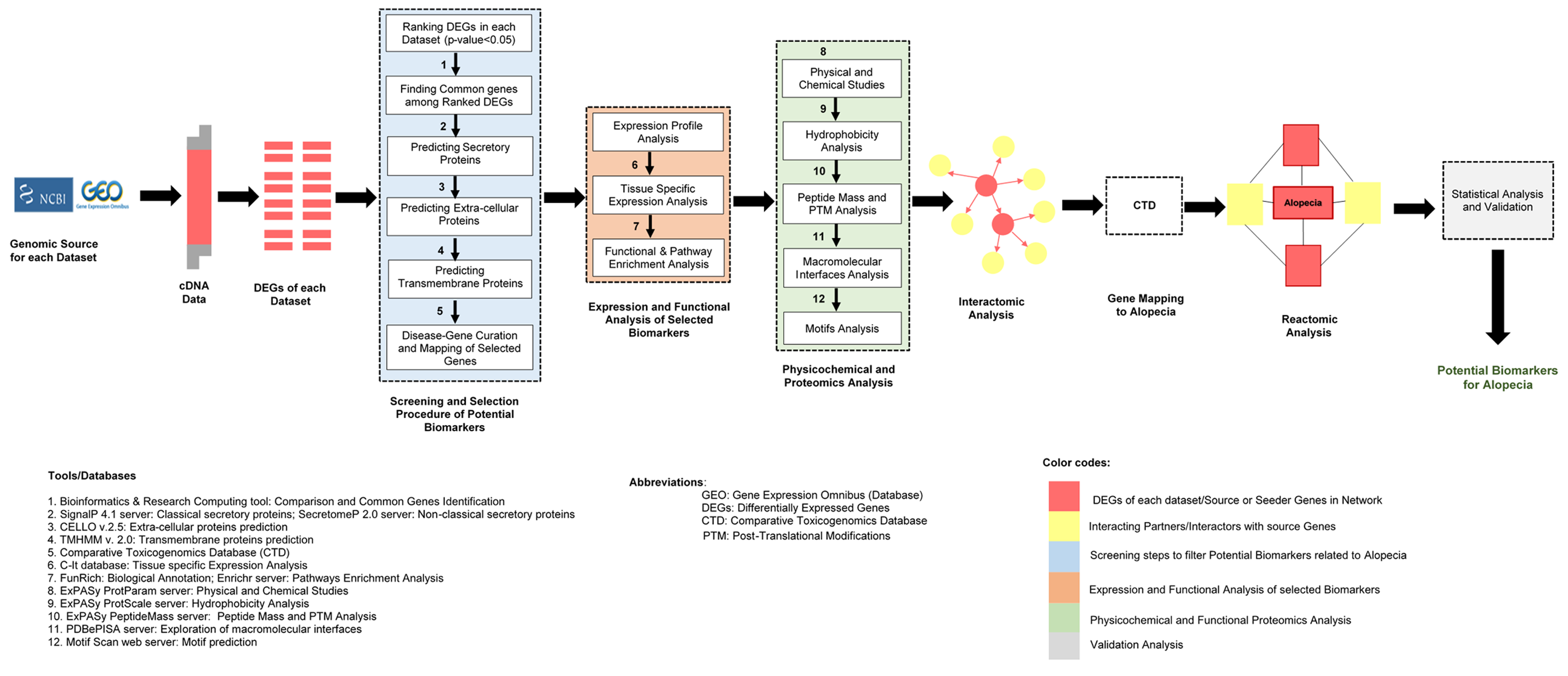

Supplement: Supplementary file 1 — Additional file 1: Fig. S1. A framework of our study designed to identify physiological biomarkers in alopecia. [file 40709_2019_94_MOESM1_ESM.tif]

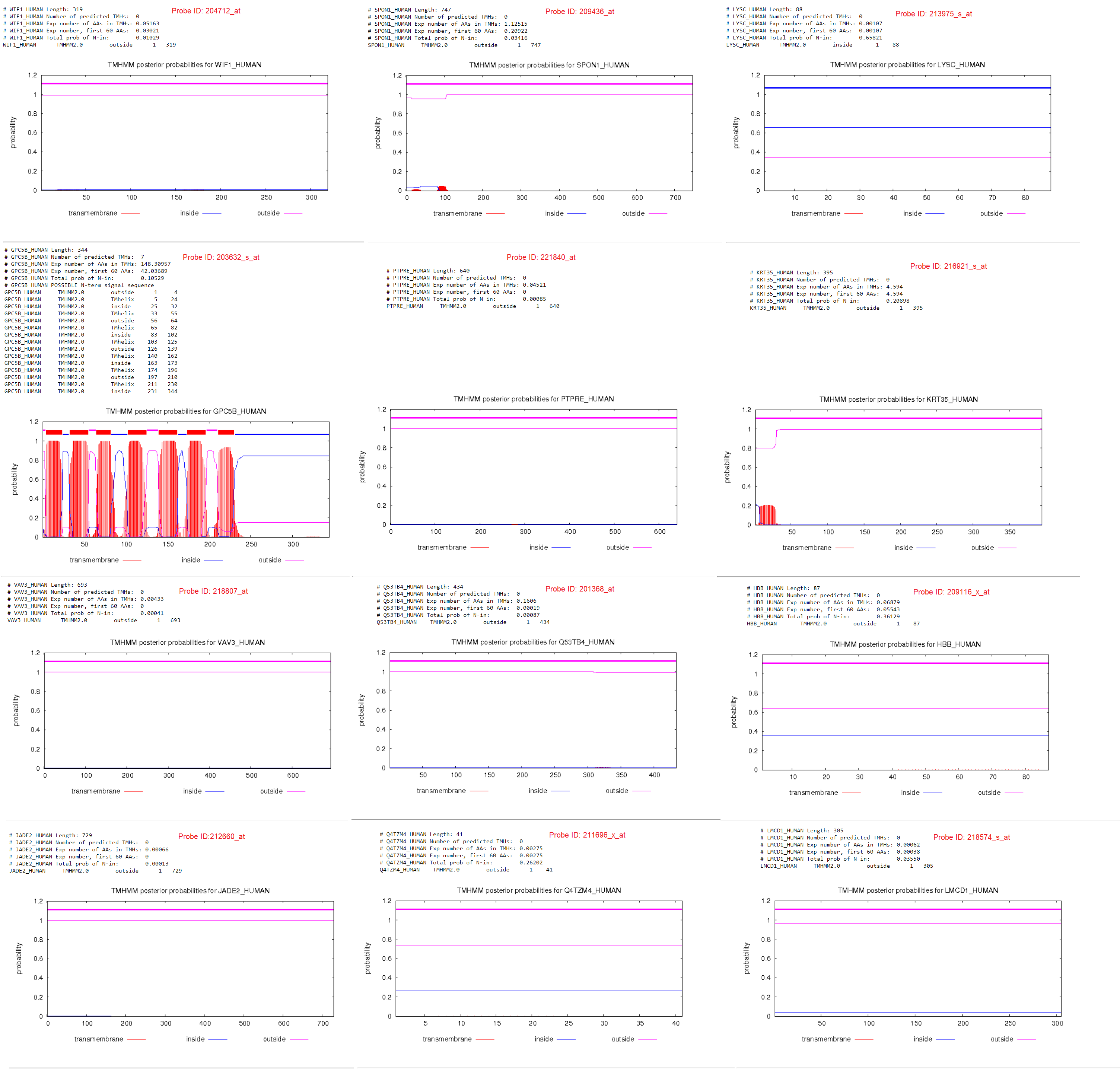

Supplement: Supplementary file 5 — Additional file 5: Fig. S2. Prediction of transmembrane helices in selected potential biomarker proteins using TMHMM Server v. 2.0. [file 40709_2019_94_MOESM5_ESM.tif]

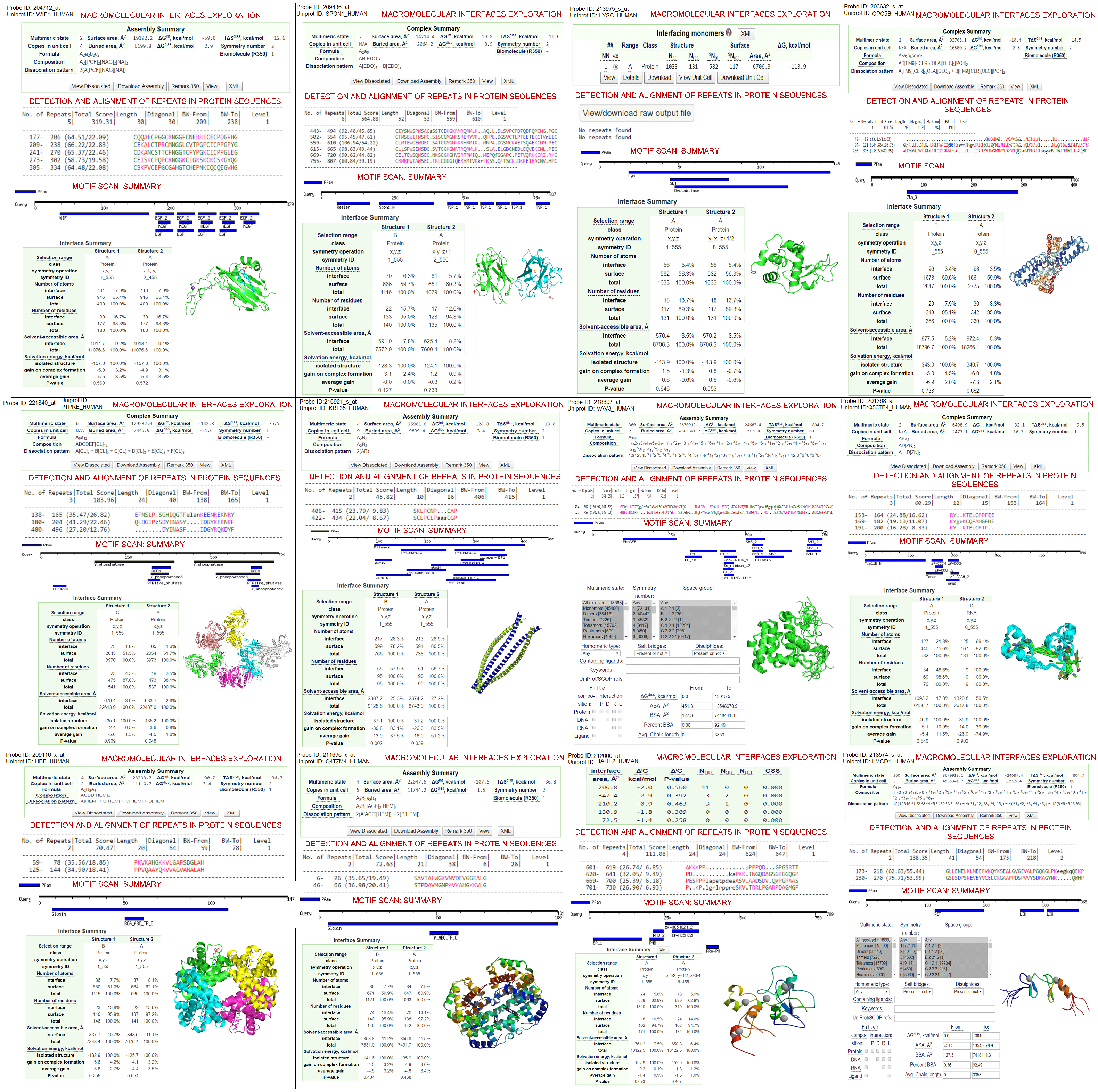

Supplement: Supplementary file 6 — Additional file 6: Fig. S3. Structural and functional properties of potential biomarker candidates. Studies of macromolecular interfaces using PDBePISA interactive tool. Motif scan in protein sequences were studied using Motif tool. Identified gapped approximate repeats and complex repeat architectures using RADAR (Rapid Automatic Detection and Alignment of Repeats) tool. [file 40709_2019_94_MOESM6_ESM.tif]

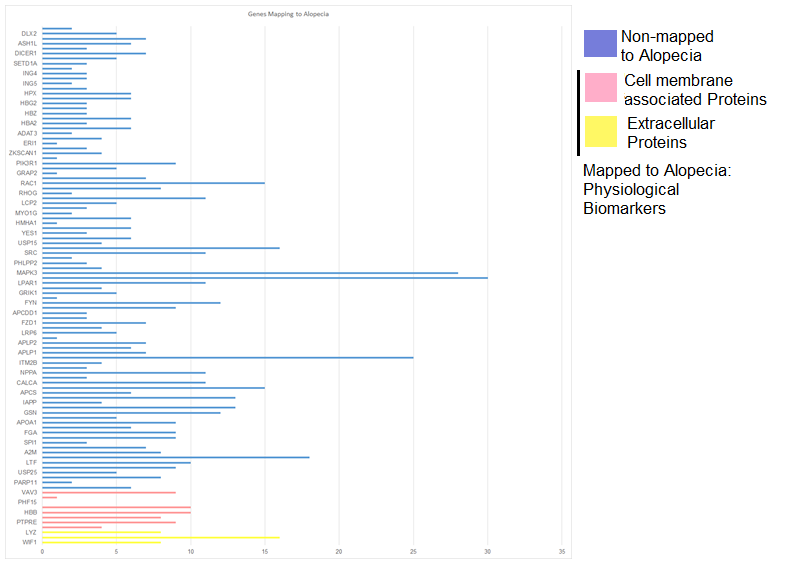

Supplement: Supplementary file 7 — Additional file 7: Fig. S4. Data mapping: The role of differentially expressed genes in alopecia was mapped using the Comparative Toxicogenomics Database (CTD). [file 40709_2019_94_MOESM7_ESM.tif]
