# Supplementary material for: A systematic simulation-based meta-analytical framework for prediction of physiological biomarkers in alopecia
Source: J Biol Res (Thessalon). 2019 Apr 4;26:2. doi: 10.1186/s40709-019-0094-x (PMC6449998; doi:10.1186/s40709-019-0094-x)
Supplement: Supplementary file 2 — Additional file 2: Table S1. The function summaryAffyRNAdeg of Bioconductor package produced a single summary-statistic for each array in the batch dataset. [file 40709_2019_94_MOESM2_ESM.docx]

**Additional file 2: Table S1**. The function summaryAffyRNAdeg of Bioconductor package produced a single summary-statistic for each array in the batch dataset.

| **Datasets** | **Parameters** | **Total Samples** | | | | | |
| --- | --- | --- | --- | --- | --- | --- | --- |
|  |  | **GSM538736.CEL** | **GSM538737.CEL** | **GSM538738.CEL** | **GSM538739.CEL** | **GSM538740.CEL** | **GSM538741.CEL** |
| GSE21569 | slope | 7.98E+00 | 6.89E+00 | 9.02E+00 | 5.49E+00 | 6.48E+00 | 6.70E+00 |
|  | p-value | 1.11E-14 | 9.91E-14 | 1.42E-14 | 5.34E-12 | 4.79E-15 | 6.40E-13 |
| GSE45512 |  | **GSM1105869.CEL** | **GSM1105870.CEL** | **GSM1105871.CEL** | **GSM1105872.CEL** | **GSM1105873.CEL** | **GSM1105874.CEL** |
|  | slope | 5.57E+00 | 4.62E+00 | 4.88E+00 | 5.04E+00 | 4.44E+00 | 5.00E+00 |
|  | p-value | 1.61E-11 | 2.82E-09 | 1.24E-09 | 6.23E-09 | 3.94E-08 | 5.52E-09 |
|  |  | **GSM1105875.CEL** | **GSM1105876.CEL** | **GSM1105877.CEL** | **GSM1105878.CEL** | - | - |
|  | slope | 4.82E+00 | 6.03E+00 | 7.87E+00 | 4.41E+00 | - | - |
|  | p-value | 5.99E-09 | 3.90E-10 | 4.88E-12 | 1.25E-08 | - | - |
| GSE36169 |  | **GSM882149.CEL** | **GSM882150.CEL** | **GSM882151.CEL** | **GSM882152.CEL** | **GSM882153.CEL** | **GSM882154.CEL** |
|  | slope | 1.75E+00 | 2.40E+00 | 2.30E+00 | 2.19E+00 | 2.34E+00 | 2.33E+00 |
|  | p-value | 1.49E-07 | 3.19E-10 | 8.88E-09 | 1.32E-08 | 2.50E-09 | 5.55E-09 |
|  |  | **GSM882155.CEL** | **GSM882156.CEL** | **GSM882157.CEL** | **GSM882158.CEL** | - | - |
|  | slope | 2.01E+00 | 2.39E+00 | 3.01E+00 | 3.32E+00 | - | - |
|  | p-value | 1.95E-07 | 9.20E-09 | 2.41E-11 | 1.81E-11 | - | - |
| GSE58573 |  | **GSM1414312.CEL** | **GSM1414313.CEL** | **GSM1414314.CEL** | **GSM1414315.CEL** | **GSM1414316.CEL** | **GSM1414317.CEL** |
|  | slope | 9.20E+00 | 8.45E+00 | 7.02E+00 | 6.90E+00 | 6.70E+00 | 5.77E+00 |
|  | p-value | 1.79E-12 | 1.79E-12 | 2.24E-12 | 4.79E-13 | 7.53E-12 | 5.28E-11 |
|  |  | **GSM1414318.CEL** | **-** | - | - | - | - |
|  | slope | 7.07E+00 | - | - | - | - | - |
|  | p-value | 4.53E-12 | - | - | - | - | - |
| GSE41680 |  | **GSM1022408.CEL** | **GSM1022409.CEL** | **GSM1022410.CEL** | **GSM1022411.CEL** | **GSM1022412.CEL** | **GSM1022413.CEL** |
|  | slope | 2.74E+00 | 2.24E+00 | 1.62E+00 | 1.09 | 2.75E+00 | 1.55E+00 |
|  | p-value | 2.88E-09 | 6.83E-07 | 8.62E-06 | 0.00281 | 3.12E-09 | 4.84E-06 |
|  |  | **GSM1022414.CEL** | **GSM1022415.CEL** | - | - | - | - |
|  | slope | 3.73E+00 | 1.15 | - | - | - | - |
|  | p-value | **9.48E-09** | **0.000596** | - | - | - | - |
| GSE3058 |  | **GSM67118.CEL** | **GSM67120.CEL** | **GSM67123.CEL** | **GSM67124.CEL** | **GSM67126.CEL** | **GSM67129.CEL** |
|  | slope | 3.54E+00 | 3.46E+00 | 3.23E+00 | 2.98E+00 | 3.20E+00 | 3.29E+00 |
|  | p-value | 1.95E-11 | 8.72E-12 | 2.06E-12 | 1.09E-10 | 1.83E-10 | 1.99E-12 |
|  |  | **GSM67131.CEL** | **GSM67132.CEL** | **GSM67134.CEL** | **GSM67138.CEL** | - | - |
|  | slope | 3.41E+00 | 3.22E+00 | 2.92E+00 | 3.24E+00 | - | - |
|  | p-value | 5.67E-12 | 1.14E-12 | 3.52E-12 | 3.21E-11 | - | - |
| GSE31324 |  | **GSM776959.CEL** | **GSM776960.CEL** | **GSM776961.CEL** | **GSM776962.CEL** | **GSM776963.CEL** | **GSM776964.CEL** |
|  | slope | 5.91E+00 | 5.38E+00 | 6.00E+00 | 6.32E+00 | 4.79E+00 | 5.57E+00 |
|  | p-value | 1.48E-11 | 3.44E-12 | 1.87E-12 | 4.27E-13 | 5.86E-11 | 1.01E-11 |
|  |  | **GSM776965.CEL** | **GSM776966.CEL** | **GSM776967.CEL** | **GSM776968.CEL** | **GSM776969.CEL** | **GSM776970.CEL** |
|  | slope | 4.01E+00 | 4.37E+00 | 4.95E+00 | 5.06E+00 | 4.64E+00 | 4.58E+00 |
|  | p-value | 1.03E-08 | 2.05E-10 | 6.72E-11 | 5.81E-11 | 7.88E-11 | 1.71E-11 |
|  |  | **GSM776971.CEL** | **GSM776972.CEL** | **GSM776973.CEL** | **GSM776974.CEL** | - | - |
|  | slope | 5.26E+00 | 4.65E+00 | 5.92E+00 | 4.67E+00 | - | - |
|  | p-value | 4.29E-12 | 1.02E-10 | 3.53E-13 | 4.86E-11 | - | - |
| GSE44765 |  | **GSM1090232.CEL** | **GSM1090233.CEL** | **GSM1090234.CEL** | **GSM1090235.CEL** | **GSM1090236.CEL** | **GSM1090237.CEL** |
|  | slope | 8.48E+00 | 8.61E+00 | 8.12E+00 | 6.82E+00 | 6.93E+00 | 7.05E+00 |
|  | p-value | 1.03E-12 | 1.27E-12 | 2.06E-12 | 1.67E-10 | 2.34E-10 | 1.26E-10 |
|  |  | **GSM1090238.CEL** | **GSM1090239.CEL** | **GSM1090240.CEL** | **GSM1090241.CEL** | **GSM1090242.CEL** | **GSM1090243.CEL** |
|  | slope | 7.43E+00 | 7.16E+00 | 7.47E+00 | 7.30E+00 | 7.33E+00 | 7.09E+00 |
|  | p-value | 3.81E-10 | 2.22E-10 | 7.73E-11 | 2.02E-10 | 1.52E-10 | 1.97E-10 |
|  |  | **GSM1090244.CEL** | **GSM1090245.CEL** | **GSM1090246.CEL** | **GSM1090247.CEL** | **GSM1090248.CEL** | **GSM1090249.CEL** |
|  | slope | 7.33E+00 | 7.42E+00 | 7.73E+00 | 6.71E+00 | 7.25E+00 | 6.41E+00 |
|  | p-value | 1.88E-10 | 1.32E-10 | 1.06E-10 | 1.21E-10 | 6.89E-11 | 1.14E-10 |
