# Supplementary material for: A systematic simulation-based meta-analytical framework for prediction of physiological biomarkers in alopecia
Source: J Biol Res (Thessalon). 2019 Apr 4;26:2. doi: 10.1186/s40709-019-0094-x (PMC6449998; doi:10.1186/s40709-019-0094-x)
Supplement: Supplementary file 3 — Additional file 3: Table S2. List of Databases, Software, and Tools used in this study. [file 40709_2019_94_MOESM3_ESM.docx]

**Additional file 3: Table S2**. List of Databases, Software, and Tools used in this study

| **Serial No.** | **Name** | **Purpose** | **Accessibility/Reference** |
| --- | --- | --- | --- |
| 1 | NCBI: Gene Expression Omnibus database | cDNA microarray datasets | http://www.ncbi.nlm.nih.gov/geo |
| 2 | R-software | Statistical analytical platform | http://www.r-project.org |
| 3 | BioConductor packages | Biological analytical software | http://www.bioconductor.org |
| 4 | Bioinformatics & Research Computing tool | Gene overlap and comparison analysis | http://jura.wi.mit.edu/bioc/tools/compare.php |
| 5 | SignalP 4.1 | Prediction of classical secretory proteins | Petersen et al., 2011 |
| 6 | CELLO v.2.5 | Sub-cellular localization prediction | Lu et al., 2004 |
| 7 | UniProt KB database | For universal protein databases | http://www.uniprot.org/ |
| 8 | SecretomeP 2.0 server | Prediction of non-classical secretory protein | Bendtsen et al., 2004 |
| 9 | TMHMM v. 2.0 | Prediction of transmembrane proteins | Moller et al., 2001 |
| 10 | Comparative Toxicogenomics Database (CTD) | Disease-Genes mapping | http://ctdbase.org/ |
| 11 | C-It database | Prediction of tissue specific genes expression | Gellert et al., 2010 |
| 12 | FunRich tool | Functional Annotation | Chen et al., 2013 |
| 13 | Enrichr tool | Pathways Enrichment | Pathan et al., 2015 |
| 14 | ExPASy ProtParam | Calculation of physicochemical parameters | Gasteiger et al., 2005 |
| 15 | ExPASy ProtScale | Hydrophobicity analysis | Gasteiger et al., 2005 |
| 16 | ExPASy PeptideMass | Calculation of peptides mass and PTM modifications | Gasteiger et al., 2005 |
| 17 | PDBePISA tool | Prediction of macromolecular interfaces | Krissinel and Henrick, 2007 |
| 18 | Motif Scan server | Prediction of motif in proteins sequences | Pagni et al., 2007 |
| 19 | RADAR tool | For gapped approximate repeats in protein | Heger and Holm, 2000 |
| 20 | SAPS tool | For compositional analysis of protein | McWilliam et al., 2013 |
| 21 | String Database | For Protein interactors | Szklarczyk et al., 2011 |
| 22 | HAPPI Database | For Protein interactors | Chen et al., 2009 |
| 23 | Cytoscape software | For PPI network visualization | Cline et al., 2007 |
| 24 | microRNA tool | For miRNAs target genes prediction | http://www.microrna.org |
| 25 | KEGG | Pathways database | http://www.genome.jp/kegg/pathway.html |
| 26 | Reactome | Pathways database | http://www.reactome.org |
| 27 | Wiki pathways | Pathways database | http://www.wikipathways.org |
| 28 | PathVisio tool | Pathways network modeling | Kutmon et al., 2015 |
