# Supplementary material for: A systematic simulation-based meta-analytical framework for prediction of physiological biomarkers in alopecia
Source: J Biol Res (Thessalon). 2019 Apr 4;26:2. doi: 10.1186/s40709-019-0094-x (PMC6449998; doi:10.1186/s40709-019-0094-x)
Supplement: Supplementary file 4 — Additional file 4: Table S3. Preliminary investigation of common and ranked differentially expressed genes of each microarray dataset. [file 40709_2019_94_MOESM4_ESM.docx]

**Additional file 4: Table S3**. Preliminary investigation of common and ranked differentially expressed genes of each microarray dataset

| **S.NO.** | **Common Probes** | **Gene Symbol** | **Uniprot_ID** | **Protein Name** | **Sub-cellular Localization** | **Secretory Proteins (SP)** |
| --- | --- | --- | --- | --- | --- | --- |
| 1 | 204712_at | WIF1 | WIF1_HUMAN | WNT inhibitory factor 1 | Extracellular | SP='YES' D=0.947 D-cutoff=0.450 |
| 2 | 209436_at | SPON1 | SPON1_HUMAN | Spondin 1, extracellular matrix protein | Extracellular | SP='YES' D=0.713 D-cutoff=0.450 |
| 3 | 213975_s_at | LYZ | LYSC_HUMAN | lysozyme (renal amyloidosis) | Extracellular | SP='YES' D=0.857 D-cutoff=0.450 |
| 4 | 203632_s_at | GPRC5B | GPC5B_HUMAN | G protein-coupled receptor | Plasma Membrane | SP='YES' D=0.764 D-cutoff=0.450 |
| 5 | 221840_at | PTPRE | PTPRE_HUMAN | protein tyrosine phosphatase | Cytoplasmic | SP='YES' D=0.648 D-cutoff=0.500 |
| 6 | 201368_at | ZFP36L2 | Q53TB4_HUMAN | zinc finger protein 36, | Nuclear | SP='NO' D=0.138 D-cutoff=0.450 |
| 7 | 209116_x_at | HBB | HBB_HUMAN | Hemoglobin, beta | Cytoplasmic | SP='NO' D=0.114 D-cutoff=0.450 |
| 8 | 211696_x_at | HBB | Q4TZM4_HUMAN | Hemoglobin-beta | Mitochondrial | SP='NO' D=0.120 D-cutoff=0.450 |
| 9 | 212660_at | PHF15 | JADE2_HUMAN | PHD finger protein 15 | Nuclear | SP='NO' D=0.102 D-cutoff=0.450 |
| 10 | 218574_s_at | LMCD1 | LMCD1_HUMAN | LIM and cysteine-rich domains 1 | Nuclear | SP='NO' D=0.123 D-cutoff=0.450 |
| 11 | 216921_s_at | KRT35 | KRT35_HUMAN | keratin 35 | Nuclear | SP='NO' D=0.118 D-cutoff=0.450 |
| 12 | 218807_at | VAV3 | VAV3_HUMAN | vav 3 guanine nucleotide exchange factor | Cytoplasmic | SP='NO' D=0.141 D-cutoff=0.450 |
